# Creep Characteristics and Damage Constitutive Model of White Sandstone Under Short-Term Freeze–Thaw Cycles

**DOI:** 10.3390/ma19102150

**Published:** 2026-05-20

**Authors:** Hepeng Zhang, Yanda Li, Peng Zeng, Kui Zhao, Dekang Shen, Xianda Yang

**Affiliations:** 1 School of Mining Engineering, Jiangxi University of Science and Technology, Ganzhou 341000, China; zhanghepengmail@163.com (H.Z.); l3027137354@163.com (Y.L.); dekangs@163.com (D.S.); xdy141516@gmail.com (X.Y.); 2 Jiangxi Province Key Laboratory of Safe and Efficient Mining of Rare Metal Resources, Ganzhou 341000, China

**Keywords:** short-term freeze–thaw, creep characteristics, acoustic emission (AE), damage mechanics, constitutive model

## Abstract

Rock masses in short-term freeze–thaw zones tend to fail under long-term loading. Therefore, investigating the creep damage characteristics of rocks under short-term freeze–thaw cycles is of great significance for the stability evaluation of rock engineering. In this study, white sandstone was used as the research material. Multi-gradient short-term freeze–thaw cycle tests and graded loading creep acoustic emission (AE) tests were performed to investigate the creep behavior and AE response characteristics of sandstone after short-term freeze–thaw action, and a creep damage constitutive model was established. The results show the following: (1) The mass loss rate, P-wave velocity reduction rate, and porosity growth rate of sandstone increase with increasing freeze–thaw cycles and duration. (2) The instantaneous axial strain of specimens increases with the stress level under different freeze–thaw durations and cycle numbers. (3) The cumulative AE event rate decreases significantly with increasing freeze–thaw cycles and duration. (4) Based on the seven-element viscoelastic model, a creep damage constitutive model was developed by introducing the freeze–thaw damage factor (D), with an average goodness-of-fit of 0.964. The findings can provide a theoretical reference for the long-term stability assessment and disaster early warning of geotechnical engineering in short-term freeze–thaw regions.

## 1. Introduction

China ranks third globally in permafrost area, which is primarily categorized into permafrost regions, seasonal frozen soil regions, and short-term cyclic freeze–thaw (CFT) regions [1]. Among these, short-term CFT regions are characterized by core features of short freezing duration, frequent temperature fluctuations, and high frequency of CFT cycles [2]. Under the combined action of short-term CFT and long-term loading, rock masses in this region are prone to instability, triggering slope collapse, large surrounding rock deformation, and other geological hazards. For example, the severe ice storm that hit southern China in 2008 affected more than 20 provinces, municipalities, and autonomous regions, and triggered widespread geological disasters. A large number of these disasters were closely linked to rock creep degradation induced by short-term freeze–thaw action [3]. Therefore, revealing the damage creep characteristics of rocks under short-term CFT is of certain reference and application value for safeguarding the long-term stability of relevant geotechnical engineering.

Scholars have confirmed through numerous experiments that cyclic freeze–thaw (CFT) is a key factor driving the degradation of rock physical and mechanical properties [4,5,6]. Increasing frequency of CFT cycles directly reduces the uniaxial compressive strength of rocks, while increasing peak strain and creep deformation, which transforms rock masses from brittle to ductile [6]. El-Garhy et al. [7] monitored the freeze–thaw process of sedimentary rocks in real time via CT scanning, clarifying that the frost-heaving effect of pore water freezing is a core factor for fracture propagation, and the damage degree is strongly correlated with pore size distribution—providing direct evidence for the study of meso-damage mechanisms. JIANG et al. [8], focusing on differences in freeze–thaw environments, found that the porosity growth rate of slate under water-freezing conditions is approximately 1.8 times that under air-freezing conditions, revealing the regulatory role of water state in freeze–thaw degradation. In summary, most existing studies focus on long-term freeze–thaw or a single freeze–thaw parameter, but lack research on the mechanical properties of rocks with “high frequency–short duration” in short-term CFT regions.

Acoustic Emission (AE) technology, as an effective method for capturing internal micro-fractures of rock masses, has been widely applied in mechanical tests of rocks after cyclic freeze–thaw (CFT) [9,10,11,12,13]. Existing studies have shown that AE signal characteristics are synchronized with the damage evolution of rock masses, and the cumulative AE event count and energy increase stepwise with increasing load, which can quantitatively reflect the cumulative degree of micro-fractures [9]. In addition, CFT-induced damage directly affects the evolution law of AE signals. With the increase in the frequency of CFT cycles, the cumulative AE event rate decreases significantly, indicating that internal fractures of rock masses have developed in advance during the CFT stage, and the concentration of fractures during the loading process is weakened [10]. Of particular importance, some studies have realized the combination of AE technology and meso-characterization techniques: ZHANG et al. [14], through synchronous CT scanning and AE monitoring, found that the pore-throat expansion rate of rock masses after CFT is positively correlated with the AE event rate, establishing a quantitative correlation between meso-pore evolution and macro-AE response. In summary, however, most existing studies focus on a single freeze–thaw duration or cycle frequency, lacking systematic analysis of the evolution law of AE signals under the “duration–frequency” coupling effect.

Taking typical white sandstone in short-term cyclic freeze–thaw (CFT) regions as the research object, this study first determines the water–ice phase transition completion point and target temperature stable freezing point of the sandstone through freeze–thaw pre-tests. Taking these two key characteristic points as control benchmarks, graded loading creep tests are designed for the sandstone under different freeze–thaw durations and cycle frequencies, with synchronous monitoring of AE signals during the rock creep failure process. It focuses on analyzing the effects of short-term CFT on the physical properties (mass, P-wave velocity, porosity), creep deformation characteristics (instantaneous strain, long-term strength), and AE responses (event rate, cumulative ringing count, *Ib* value) of the sandstone. Finally, based on the seven-element viscoelastic model, a CFT-induced damage factor is introduced to construct a creep damage constitutive model. The research findings are expected to provide a reference basis for the long-term stability evaluation and disaster prevention of geotechnical engineering in short-term CFT regions.

## 2. Experimental Introduction

### 2.1. Specimens Preparation

The white sandstone used in this test was collected from Pingshan County, Yibin City, Sichuan Province, China (E 103°56′, N 28°41′). It is a thick-bedded pale white medium–fine grained quartz sandstone with uniform texture and good integrity. The sampling depth ranges from 0.5 to 1.0 m below the surface outcrop, featuring low weathering degree and weakly developed joints. According to the permafrost regionalization data from the National Cryosphere Science Data Center [15], the sampling site is located in the seasonal short-term frozen soil zone. The specimens are grayish-white, homogeneous, and all taken from the same parent rock. In accordance with ISRM standards [16], the rock specimens were processed into standard cylinders with a height of 100 mm and a diameter of 50 mm. The specimen preparation fit the following precision requirements: the parallelism error of the end faces ≤ ±0.02 mm; the perpendicularity deviation between the end faces and the specimens axis ≤ ±0.25°. Sandstone specimens with similar mass and P-wave velocity were selected for subsequent tests.

### 2.2. Main Experimental Equipment and Scheme

Most scholars adopt a fixed freeze–thaw duration (usually ≥3~4 h) when designing cyclic freeze–thaw (CFT) tests to ensure complete freezing and thawing of rocks. After summarizing relevant domestic and international CFT test literatures, SHEN et al. [17] concluded that the complete freezing time of common rocks is 2 h and the thawing time is 4 h, with the specific duration decreasing as rock porosity increases. Some scholars use shorter freeze–thaw durations in their research, but none have clarified the basis for setting the freeze–thaw duration or the key time nodes. Therefore, pre-tests were conducted to determine the basis for setting the freeze–thaw duration and the critical time nodes.

(1) Freeze–Thaw Cycle Pre-Test

**Figure 1:**
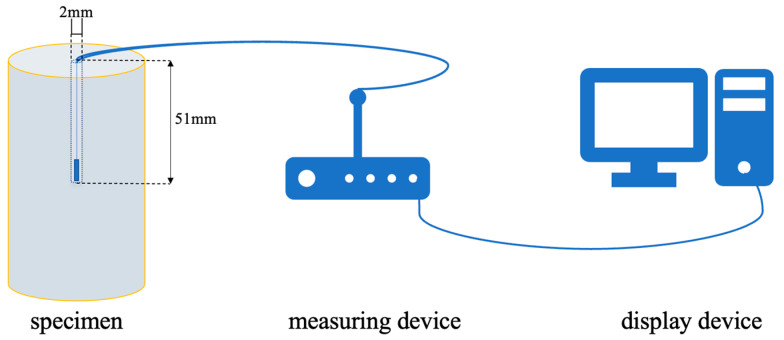
Measurement of temperature in the center of specimens.

Firstly, a hole is drilled at the center of one end face of the specimen. After water saturation (Hebei Yinfeng Experimental Instrument Co., Ltd., Cangzhou, Hebei, China), a thermistor temperature sensor is inserted into the core of the specimen. The real-time temperature data are recorded using a dual-channel thermometer (Model: YET-720, Shenzhen YOWEXA Sensor System Co., Ltd., Shenzhen, China), with the sampling frequency of the thermometer set to 1 sample per minute. Secondly, the drilled hole is sealed with 704 adhesive; after the adhesive cures, the specimen is immersed in water to test the sealing tightness of the sealed hole. The schematic diagram of the central temperature measurement of the specimen is shown in Figure 1. After repeated confirmation that the tightness is qualified, the specimen is placed in a freeze–thaw chamber (Danble Instruments Co., Ltd., Kunshan, Jiangsu, China). Referring to previous research results [18,19], the temperature and duration are designed as −10 °C for 4 h of freezing and 20 °C for 4 h of thawing to ensure complete freeze–thaw. The central temperature of the specimen is measured to determine the time required for the water–ice phase transition completion point and the target temperature complete freezing point of the specimen, providing a basis for the design of subsequent freeze–thaw durations.

**Figure 2:**
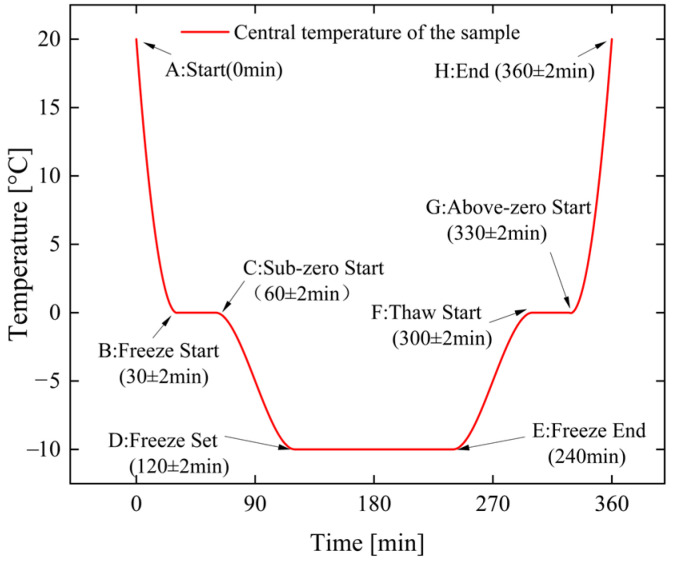
Sandstone center temperature–time curve.

Based on the above, the statistical results of the variation law of the white sandstone center temperature with time are shown in Figure 2. It can be seen from the figure that the variation process of the sandstone center temperature can be divided into the following stages:

① Freezing stage

During AB, the central temperature of the sandstone drops to the water–ice phase transition point (30 ± 2 min); during BC, the sandstone undergoes water–ice phase transition, and freezing initiates (30 ± 2 min); during CD, the central temperature of the sandstone continues to drop to the target temperature (60 ± 2 min). After point D, the central temperature of the rock remains stable near the target temperature.

② Thawing stage

During EF, the central temperature of the sandstone rises to the water–ice phase transition point (60 ± 2 min); during FG, the sandstone undergoes water–ice phase transition, and thawing initiates (30 ± 2 min); during GH, the central temperature of the sandstone continues to rise to the target temperature (30 ± 2 min); after point G, the central temperature of the rock remains stable near the target temperature.

It can be concluded from the above that when the freezing duration is 1 h, the water–ice phase transition inside the specimen has been completed and the rock has been frozen; when the freezing duration is 2 h, the central temperature of the rock has stabilized and the rock is in a stable frozen state. Therefore, these two cyclic freeze–thaw (CFT) durations—1 h freezing/1 h thawing and 2 h freezing/2 h thawing—are selected for subsequent CFT tests, so as to reveal the damage evolution mechanism of white sandstone under short-term CFT.

(2) Experimental Scheme

**Figure 3:**
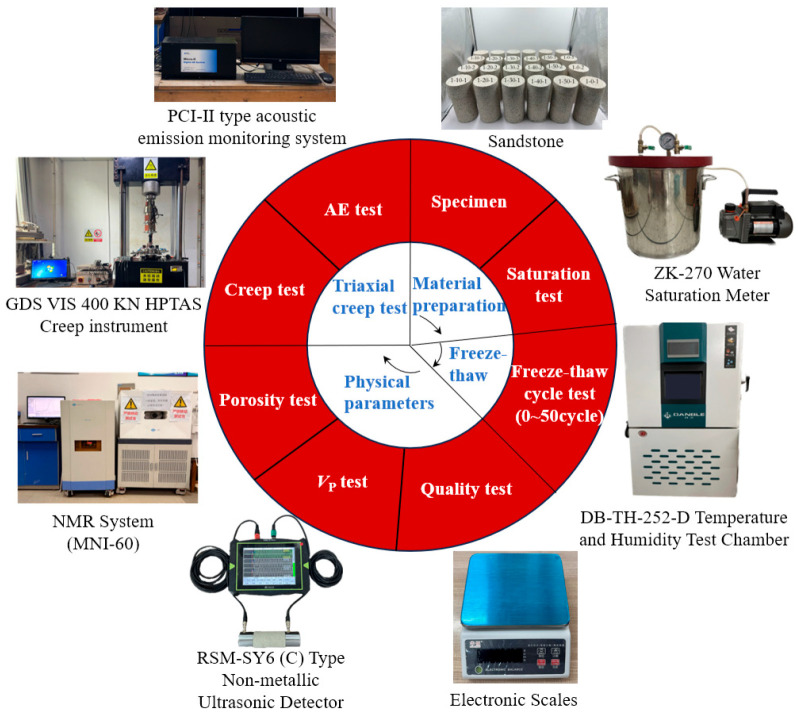
Main experimental equipment and flow.

All specimens were subjected to water saturation treatment. Two freeze–thaw cycle working conditions were set: 1 h freezing + 1 h thawing and 2 h freezing + 2 h thawing. The number of freeze–thaw cycles was designed as 0, 10, 20, 30, 40, and 50 times, respectively. Meanwhile, a group of untreated specimens was arranged as the control group. During the freeze–thaw cycles, an electronic balance (manufacturer: Shanghai Sunny Hengping Scientific Instrument Co., Ltd., Shanghai, China), RSM-SY6(C) nonmetallic ultrasonic detector(manufacturer: Wuhan Sinorock Technology Co., Ltd., Wuhan, China) and NMR system (manufacturer: Suzhou Niumag Analytical Instrument Co., Ltd., Suzhou, China)were adopted to measure the mass, longitudinal wave velocity, and porosity of each group of specimens before and after every freeze–thaw cycle. After the completion of all freeze–thaw cycles, graded loading creep tests were carried out (GDS Instruments Ltd., Hook, Hampshire, UK), and acoustic emission (AE) signals were collected synchronously during the tests. The acoustic emission test parameters were set as follows: the threshold value was 40 dB, the preamplifier gain was 40 dB, the sampling length was 2 k, the sampling frequency was 2 MS/s, and the sensor model was R15a (Manufacturer: Physical Acoustics Corporation of the United States, a subsidiary of the MISTRAS Group, located in Princeton, NJ, USA). The whole experimental procedure is shown in Figure 3.

The graded loading stress levels of the creep tests were determined based on the uniaxial compressive strength (*R_c_*) of specimens under the same conditions. Specifically, 30% *R_c_*, 40% *R_c_*, 50% *R_c_*, 60% *R_c_*, 70% *R_c_*, and 80% *R_c_* were selected as the loading stress levels for grades 1 to 6 in the creep tests, respectively. Combined with the research paradigm of creep tests on similar sandstones and the results of preliminary pre-tests, the load holding time under all loading levels is uniformly set to 10 h [18]. When the loading stress exceeds 80% *R_c_*, to avoid instantaneous loading failure of the specimens, the subsequent stress increment per level was adjusted to 5% *R_c_*, and 85% *R_c_* was used as the loading stress level for grade 7 in the creep tests until creep failure occurs. The graded loading stress levels of the creep tests are shown in Table 1. The loading control scheme of the creep tests adopted the force control mode, and the loading rate in the instantaneous loading stage (where stress jumps to the target value) was 0.2 kN/s.

## 3. Variation of Physical and Mechanical Properties in Sand

### 3.1. Characteristics of Physical Parameter Changes

Physical parameters of rocks (mass, P-wave velocity, porosity) are basic indicators reflecting their internal structural state.

Equation (1) was used to calculate the mass change rate ($\eta_{m}$), P-wave velocity change rate ($\eta_{V}$), and porosity change rate ($\eta_{Q}$) of sandstone before and after freeze–thaw tests.

(1) $$\eta_{N}=\frac{N_{0}-N_{1}}{N_{0}}\times 100\%$$

where $N_{0}$ is the mass, P-wave velocity, and porosity of sandstone before freeze–thaw cycles; $N_{1}$ is the mass, P-wave velocity, and porosity of sandstone after freeze–thaw cycles; $\eta_{N}$ is the mass change rate, P-wave velocity change rate, and porosity change rate of sandstone.

**Figure 4:**
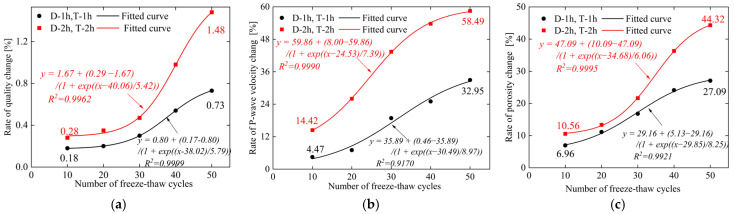
Variation laws of physical parameters of sandstone under different freeze–thaw cycles. (**a**) Quality loss rate curve; (**b**) P-wave velocity attenuation rate; (**c**) Porosity growth rate.

The data of mass, P-wave velocity, porosity, and their corresponding change rates of sandstone are shown in Figure 4.

(1) It can be seen from Figure 4a. that the mass loss rate increases with the rise in the frequency of cyclic freeze–thaw (CFT) cycles, while the magnitude of change under the 2 h freezing/2 h thawing condition is significantly larger than that under the 1 h freezing/1 h thawing condition. Under the condition of 10 cycles, extending the freeze–thaw duration by 1 h increased the mass change rate of the specimens from 0.18% to 0.28%, a 1.56-fold increase. At 50 cycles, extending the freeze–thaw duration by 1 h raised the mass change rate from 0.73% to 1.48%, representing a 2.03-fold increase. This indicates that extending the freeze–thaw duration by 1 h exacerbates the surface damage of the specimens.
(2) It can be seen from Figure 4b. that the P-wave velocity reduction rate increases with the increase in the frequency of cyclic freeze–thaw (CFT) cycles [20], while the magnitude of change under the 2 h freezing/2 h thawing condition is significantly larger than that under the 1 h freezing/1 h thawing condition. Under the condition of 10 cycles, extending the freeze–thaw duration by 1 h increased the P-wave velocity reduction rate of the specimens from 4.47% to 14.42%, a 3.23-fold increase. At 50 cycles, extending the freeze–thaw duration by 1 h raised the mass change rate from 32.95% to 58.49%, representing a 1.78-fold increase. This indicates that extending the freeze–thaw duration aggravates the internal structural damage of the specimens.
(3) It can be seen from Figure 4c. that the porosity growth rate increases with the increase in the frequency of cyclic freeze–thaw (CFT) cycles, while the magnitude of change under the 2 h freezing/2 h thawing condition is significantly larger than that under the 1 h freezing/1 h thawing condition. Under the condition of 10 cycles, extending the freeze–thaw duration by 1 h increased the mass change rate of the specimens from 6.96% to 10.56%, a 1.52-fold increase. At 50 cycles, extending the freeze–thaw duration by 1 h raised the porosity change rate from 27.09% to 44.33%, representing a 1.64-fold increase. This indicates that extending the duration of a single freeze–thaw cycle significantly aggravates the internal structural damage of the specimens [21].

### 3.2. Creep Deformation Magnitude Characteristics

**Figure 5:**
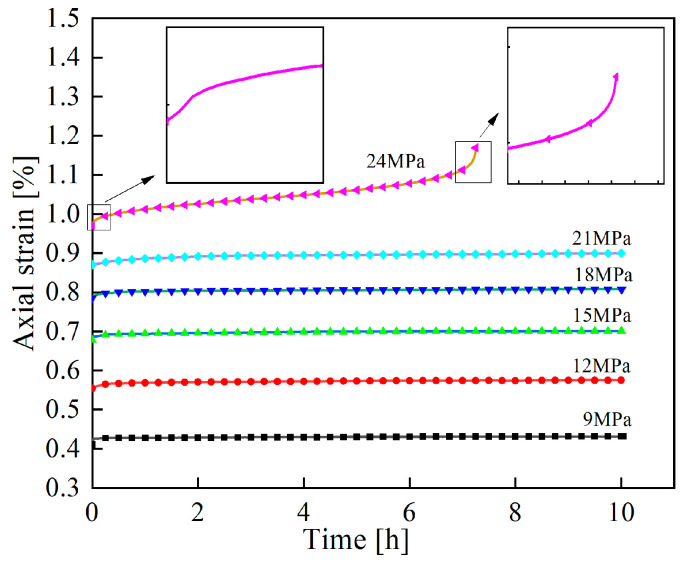
Axial strain–time creep curve of unfrozen–thawed sandstone.

Graded loading creep tests were performed on the specimens using a creep testing machine. Taking the creep curve of unfrozen–thawed specimens (Figure 5) as an example, the division of creep stages shows that the creep process can be categorized into three typical stages: axial strain increases rapidly in the decelerating creep stage; axial strain increases slowly in the steady creep stage; and axial strain increases sharply in the accelerating creep stage, which occurs only under the failure stress level, with the specimen being on the verge of unstable failure.

**Figure 6:**
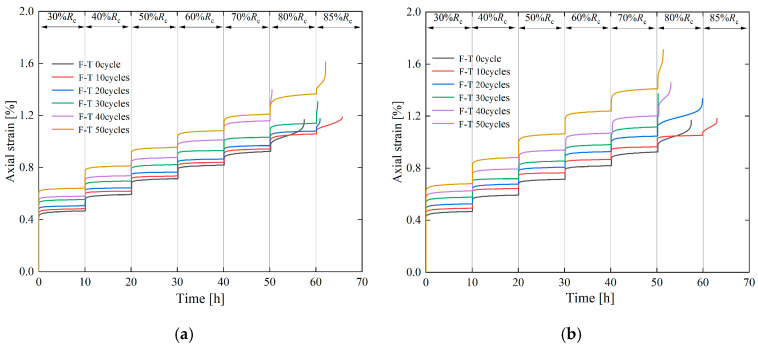
Axial strain–time creep curves of sandstone under different freeze–thaw cycle conditions. (**a**) F-1 h, T-1 h; (**b**) F-2 h, T-2 h.

The creep characteristics of specimens under different cyclic freeze–thaw (CFT) conditions are shown in Figure 6, and their creep curves all exhibit the same three-stage characteristics as those of unfrozen–thawed specimens. Under the non-failure stress levels (loading stress levels < 80% *R_c_*), only the decelerating creep stage and steady creep stage occur. Under the failure stress levels (loading stress levels ≥ 80% *R_c_*), the specimens exhibit the complete three creep stages, namely the decelerating, steady, and accelerating creep stages.

**Figure 7:**
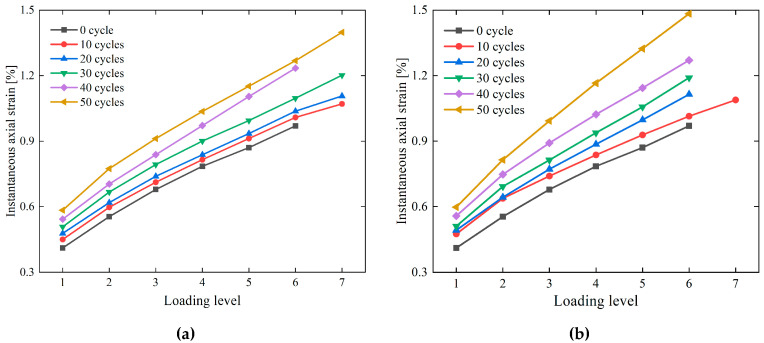
Instantaneous strain versus loading level creep curves of sandstone under different numbers of cycles. (**a**) F-1 h, T-1 h; (**b**) F-2 h, T-2 h.

Table 2 presents the data of instantaneous axial strain of specimens at each stress level under different freeze–thaw durations and cyclic freeze–thaw (CFT) cycle frequencies, with the corresponding variation law illustrated in Figure 7. Analysis indicates that under different CFT durations and cycle frequencies, the instantaneous axial strain of the specimens exhibits an increasing trend with the increase in each stress level.

It can be seen from Figure 7 that under the same stress level, the instantaneous axial strain of the specimens increases with the increase in cyclic freeze–thaw (CFT) cycle frequency. Under the condition of 1 h freezing/1 h thawing with 40 CFT cycles, the instantaneous axial strains of the specimens at stress levels 1 and 6 are 0.543 and 1.235, respectively, which are 1.32 times and 1.27 times those of the 0-cycle specimens (unfrozen–thawed) at stress level 1 (0.411) and stress level 6 (0.970). Under the condition of 2 h freezing/2 h thawing with 40 CFT cycles, the instantaneous axial strains of the specimens at stress levels 1 and 6 are 0.558 and 1.271, respectively, corresponding to 1.36 times and 1.31 times those of the 0-cycle specimens at stress level 1 (0.411) and stress level 6 (0.970).

It can be seen from Table 2 that under the same stress level, the instantaneous axial strain of the specimens increases with the extension of freeze–thaw duration. After 10 cyclic freeze–thaw (CFT) cycles, the instantaneous axial strains of the 2 h freezing/2 h thawing specimens at stress levels 1 and 6 are 0.475 and 1.014, respectively, which are 1.01 times and 1.00 times those of the 1 h freezing/1 h thawing specimens at stress level 1 (0.450) and stress level 6 (1.009). After 50 CFT cycles, the instantaneous axial strains of the 2 h freezing/2 h thawing specimens at stress levels 1 and 6 are 0.598 and 1.484, respectively, corresponding to 1.02 times and 1.17 times those of the 1 h freezing/1 h thawing specimens at stress level 1 (0.584) and stress level 6 (1.269).

Under the same cyclic freeze–thaw (CFT) cycle frequency, the instantaneous axial strain of 1 h freezing/1 h thawing specimens is consistently smaller than that of 2 h freezing/2 h thawing specimens. This difference indicates that prolonging the freeze–thaw duration facilitates more sufficient freezing of fissure water, thereby accelerating the initiation and propagation of internal fissures. Consequently, the 2 h freezing/2 h thawing specimens suffer more severe damage to their microstructure, leading to a sharp decline in their deformation resistance. In contrast, the 1 h freezing/1 h thawing specimens sustain relatively minor freeze–thaw damage and retain a relatively intact microstructure. Overall, with the extension of freeze–thaw duration and the increase in cycle frequency, internal fissures in the specimens are gradually connected, and their load-bearing capacity and deformation resistance will be further impaired.

### 3.3. Long-Term Strength

**Figure 8:**
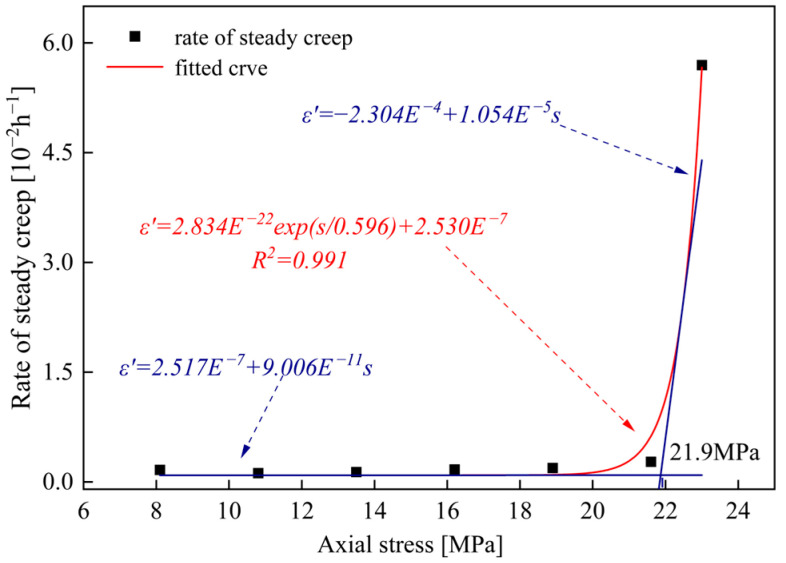
Fitting curve of steady creep rate versus axial stress for sandstone after 20 cycles of freeze-1 h and thaw-1 h.

The long-term strength of rocks is a key indicator for evaluating the stability and safety of engineering projects, and there exist diverse methods for its determination [14,22,23]. Among these methods, the steady-state creep rate method is the most commonly adopted [24]. The determination method is illustrated in Figure 8.

**Figure 9:**
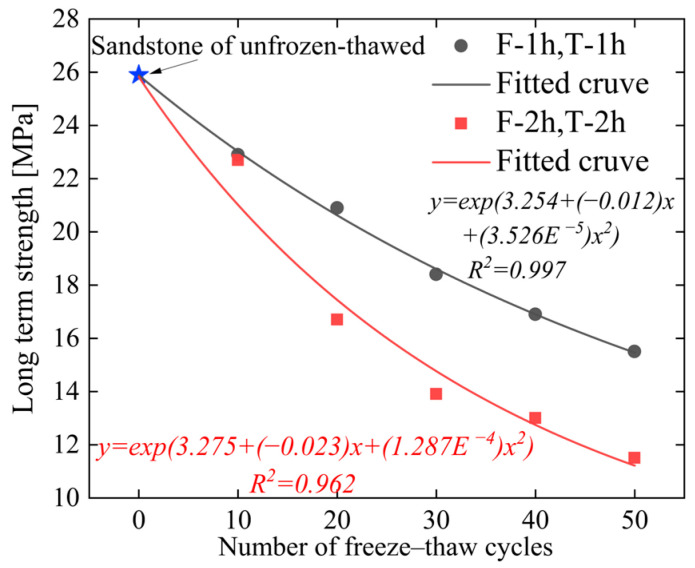
Long-term strength changes of sandstone under different cyclic freeze–thaw.

By adopting the method proposed by SUN Xiao-ming et al. [24], the long-term strength of the specimens is presented in Figure 9. The analysis shows that under the same freeze–thaw duration, the long-term strength of the specimens decreases with the increase in cyclic freeze–thaw (CFT) cycle frequency. The freeze–thaw duration directly affects the magnitude of strength reduction; the longer the freeze–thaw duration, the lower the long-term strength of the specimens after cyclic treatment. Taking 50 CFT cycles as an example, the long-term strength of the 1 h freezing/1 h thawing specimens is 15.5 MPa, while that of the 2 h freezing/2 h thawing specimens is 11.5 MPa, representing a decrease of 25.8%. This phenomenon can be attributed to the internal structural evolution characteristics of the specimens. In the initial state, the specimens exhibit a low degree of fissure development, and the frost-heaving effect induced by water–ice phase transition is relatively weak. With the extension of freeze–thaw duration, rock porosity continues to develop, water intrusion is intensified, and the frost-heaving effect is gradually enhanced, ultimately leading to a decline in the internal structural stability of the specimens. This finding is consistent with the meso-damage analysis results reported by scholars such as Alneasan et al. [25,26,27,28].

## 4. Creep Acoustic Emission Characteristics of Sandstone

Acoustic Emission (AE) technology, as a real-time monitoring technique for the rock damage evolution process, can effectively capture the energy release behavior associated with the initiation, propagation, and coalescence of internal micro-cracks in rocks [29].

### 4.1. Stress Stage Evolution Characteristics of Acoustic Emission

**Figure 10:**
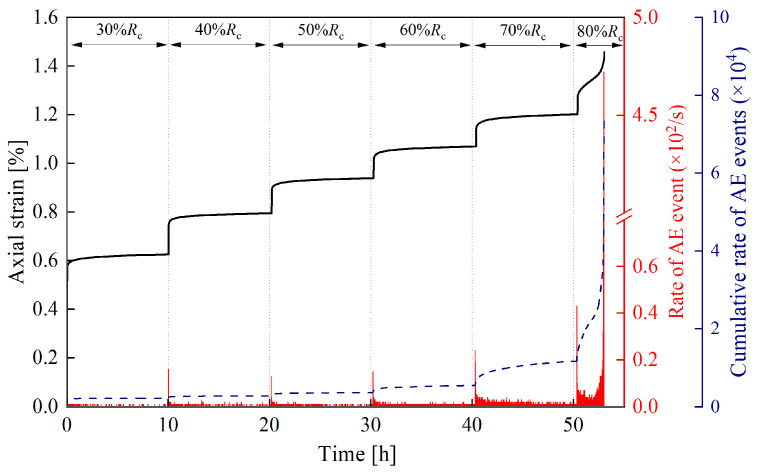
Variation curves of AE event rate and axial strain with time during the entire creep process of sandstone after 40 cycles of F-2 h and T-2 h.

The damage evolution of rocks during creep can be divided into two typical stress stages: non-destructive stress stage (loading stress level < 80% *R_c_*) and destructive stress stage (loading stress level ≥ 80% *R_c_*). Figure 10 shows the variation characteristics of AE event rate and axial strain with time during the entire creep process of sandstone after 40 cycles of F-2 h and T-2 h.

The creep acoustic emission (AE) event rate exhibits a typical three-stage evolution law of “increase–decrease–stabilization” under the non-failure stress stage [30,31]. During the instantaneous loading stage, when the stress jumps to the target value, the AE event rate rises rapidly, which reflects the instantaneous activation of existing internal micro-cracks and the rapid initiation of new cracks in the rock. In the decelerating creep stage, the AE event rate decreases gradually, indicating that the crack propagation rate slows down and the internal stress distribution of the rock tends to be balanced. In the steady creep stage, the event rate stabilizes at a low level (0–10 events/s), which suggests that rock damage enters a relatively stable state and the propagation and closure of micro-cracks achieve dynamic equilibrium.

The acoustic emission (AE) event rate exhibits a distinct U-shaped variation characteristic under the failure stress stage. During the decelerating creep stage, the AE event rate rises briefly at the initial stage and then decreases gradually. In the steady creep stage, the AE event rate remains relatively stable. In the accelerating creep stage, the AE event rate increases sharply (up to more than 1000 events/s), and the cumulative number of events rises steeply, which reflects the rapid propagation of macroscopic cracks and ultimately leads to the unstable failure of the specimens [32].

#### 4.1.1. Acoustic Emission Characteristics Under Different Freeze–Thaw Cycle Durations

**Figure 11:**
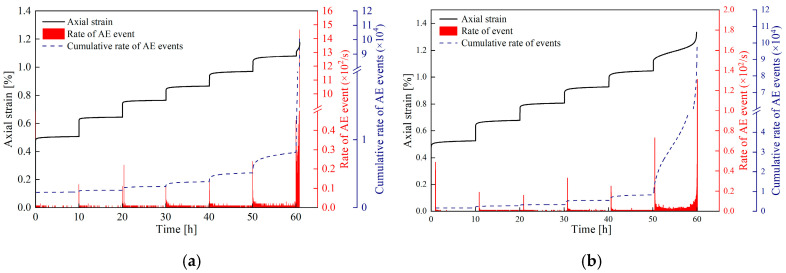
Variation curves of AE event rate and cumulative event rate of sandstone under two freeze–thaw durations after 20 freeze–thaw cycles. (**a**) F-1 h, T-1 h; (**b**) F-2 h, T-2 h.

Different freeze–thaw durations have obvious influences on the AE characteristics of white sandstone under the same number of cycles. Figure 11 shows the variation laws of AE event rate and cumulative event rate of sandstone under two different freeze–thaw durations after 20 freeze–thaw cycles.

In the non-destructive stress stage, both freeze–thaw states exhibit the common characteristic of “a sharp surge in event rate at the initial loading stage—a decrease in event rate during the decelerating creep stage—stabilization of event rate in the steady-state creep stage”, but with significant differences. For the sandstone subjected to 1 h freezing/1 h thawing, the peak event rate at the initial loading stage is relatively high (approximately 22 counts/s), and stabilizes at 0~1 counts/s during the steady-state creep stage. In contrast, the sandstone with 2 h freezing/2 h thawing shows a relatively lower peak event rate (15~18 counts/s) at the initial loading stage, accounting for about 70%~80% of that of the 1 h freezing/1 h thawing sandstone; its event rate stabilizes at 5~10 counts/s in the steady-state creep stage, with an even lower event occurrence frequency.

In the destructive stress stage, both freeze–thaw conditions exhibit U-shaped AE characteristics, but the difference in the rate of damage evolution is significant. Specifically, for the 1 h freezing/1 h thawing specimens, the peak AE event rate in the decelerating creep stage is approximately 24 events/s, and the AE event rate surges sharply to 1538 events/s in the accelerating creep stage. For the 2 h freezing/2 h thawing specimens, the peak AE event rate in the decelerating creep stage ranges from 70 to 85 events/s, and the maximum AE event rate in the accelerating creep stage is 1200 to 1300 events/s. Moreover, the moment when the AE event rate surges sharply occurs earlier than that of the 1 h freezing/1 h thawing specimens, indicating that substantial damage occurs in these specimens at a lower stress level.

The above differences can be attributed to the discrepancies in the internal microstructure of rocks under different freeze–thaw conditions. Compared with the 1 h freezing/1 h thawing specimens, the water–ice phase transition inside the 2 h freezing/2 h thawing specimens is more sufficient, and the duration of the generated frost-heaving force is longer, resulting in higher initial micro-crack density and better connectivity. Therefore, under the same stress level, its acoustic emission (AE) activity intensity is relatively lower, further indicating that substantial damage occurs at a lower stress level [33]. AE activity shows good synchrony with macroscopic creep strain, and together they reveal the entire process of rock from initial damage and stable propagation to final unstable failure.

#### 4.1.2. Acoustic Emission Characteristics Under Different Freeze–Thaw Cycle Numbers

**Figure 12:**
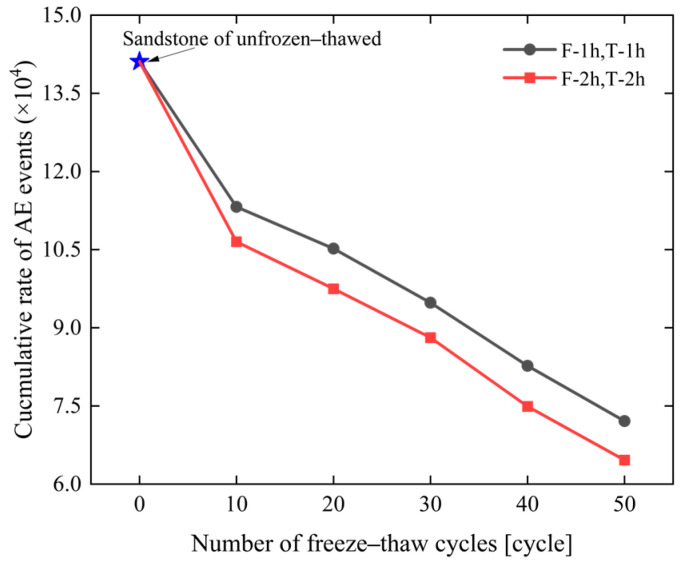
Variation curve of cumulative AE event rate of sandstone with the number of cycles under different freeze–thaw durations.

An increase in cyclic freeze–thaw (CFT) cycle frequency exerts an influence on the acoustic emission (AE) characteristics of the specimens under the same freeze–thaw duration. Figure 12 illustrates the variation law of the AE cumulative event rate of the specimens under different CFT cycle frequencies. It can be concluded from Figure 12 that with the increase in CFT cycle frequency, the AE cumulative event rates of the specimens under the two freeze–thaw conditions both show a continuous decreasing trend, but there are significant differences in the magnitude and rate of decline. For the 1 h freezing/1 h thawing specimens, the cumulative event rate decreases to 85% of that of the unfrozen–thawed specimens after 10 cycles and drops to 55% after 50 cycles, with the decline process showing a gentle linear trend. For the 2 h freezing/2 h thawing specimens, the cumulative event rate has already decreased to 70% of that of the unfrozen–thawed specimens after 10 cycles and only accounts for 30% after 50 cycles, with the decline rate slowing down significantly after 30 cycles.

The AE activity intensity of the 2 h freezing/2 h thawing specimens is consistently lower than that of the 1 h freezing/1 h thawing specimens under the same cyclic freeze–thaw (CFT). After 20 CFT cycles, the AE cumulative event rate of the 2 h freezing/2 h thawing specimens accounts for approximately 65% of that of the 1 h freezing/1 h thawing specimens; after 50 cycles, this proportion further drops to 52%.

In summary, with the increase in cyclic freeze–thaw (CFT) cycle frequency and duration, the degree of microstructural damage inside the rock intensifies and the initial crack network becomes more developed, resulting in a higher energy threshold required for the initiation of new cracks under the same stress loading conditions and thus a reduction in acoustic emission (AE) activity. This also reflects the cumulative effect of freeze–thaw damage, indicating that prolonged freeze–thaw causes more pronounced damage to the rock microstructure.

### 4.2. Evolution Characteristics of Ib Value in the Creep Acoustic Emission Failure Stage

In the field of seismic research, Gutenberg and Richter first proposed to describe the statistical relationship between earthquake magnitude and frequency through the *b*-value [34], as shown in Equation (2). Changes in the b-value are often used as precursor characteristics of seismic activity.

(2) lgN=a−bM

where *M* is the earthquake magnitude; *N* is the number of earthquakes with magnitudes between *M* and Δ*M*; a is a constant representing the degree of seismic activity; *b* is the *b*-value in seismology, which can reflect the proportional change of earthquakes with different magnitudes.

Gutenberg [34] pointed out that there is a certain similarity between the rock deformation and failure process and seismic activity. Therefore, the *b*-value is introduced into AE technology to monitor the stability of rock engineering. With the progress of research, Rao et al. [35] have improved the b-value and proposed the concept of the *Ib* value, which is considered to better characterize the degree of crack expansion inside the material. When the *Ib* value increases, it indicates that small-scale fractures dominate inside the material; when the *Ib* value decreases, it indicates that large-scale fractures dominate inside the material; when the *Ib* value is stable, it indicates that the micro-cracks inside the material are in a stable development process [18,19]. The calculation formula of the *Ib* [36] value is as follows:

(3) $$Ib=\frac{\log_{10}N(w_{1})-\log_{10}N(w_{2})}{(\alpha_{1}+\alpha_{2})\sigma }$$

where σ is the amplitude standard deviation; $\alpha_{1}$ and $\alpha_{2}$ are constants; $N(w_{1})$ is the cumulative number of AE hits with amplitude greater than $\left(\mu -\alpha_{2}\sigma \right)$; $N(w_{2})$ is the cumulative number of AE hits with amplitude greater than $\left(\mu -\alpha_{1}\sigma \right)$; μ is the average AE amplitude.

**Figure 13:**
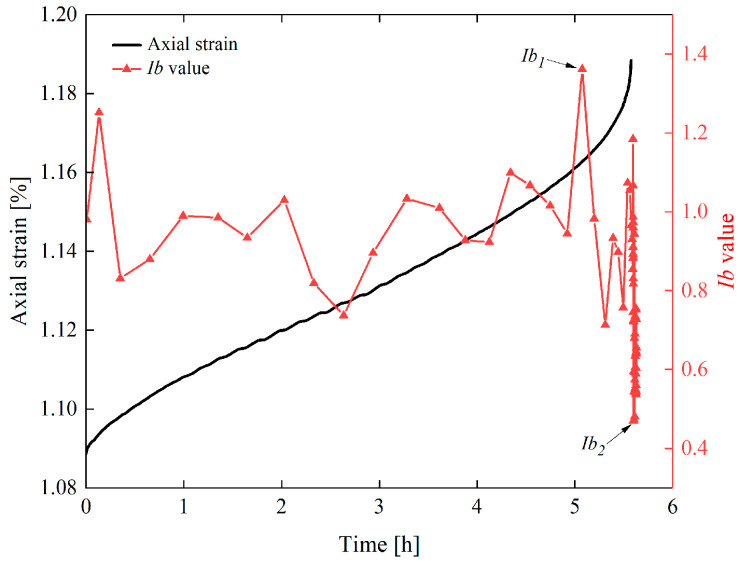
The variation curve of axial strain and *Ib* value of sandstone over time after 10 freeze–thaw cycles with F-1 h, T-1 h.

**Figure 14:**
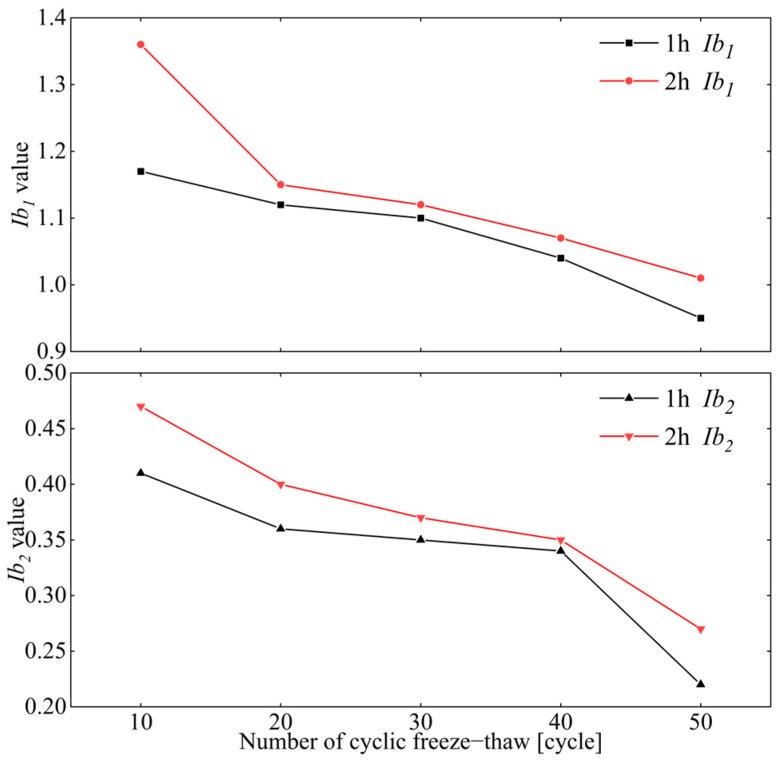
The variation curve of the number of cyclic freeze–thaw with *Ib*_1_ and *Ib*_2_ value of sandstone under different freeze–thaw times.

Taking the specimen under 10 cycles of 1 h freezing/1 h thawing as an example, Figure 13 defines the maximum value of AE *Ib* as *Ib*_1_ and the minimum value as *Ib*_2_. The specific AE *Ib* values are shown in Figure 14.

As shown in Figure 14, under the same freeze–thaw duration, the AE *Ib*_1_ and *Ib*_2_ values exhibit a continuous decreasing trend with the increase in the number of freeze–thaw cycles, indicating that the accumulation of freeze–thaw cycles significantly changes the mechanical properties and damage evolution mechanism of rocks. The essential reason is that during the freeze–thaw cycles, the repeated frost heaving–thawing shrinkage induced by water–ice phase transition acts continuously on the internal pore walls of rocks, promoting the continuous propagation and coalescence of initial micro-defects and the continuous initiation of new micro-pores and micro-cracks. With the increase in the number of cycles, the cumulative effect of micro-defects gradually becomes prominent, the connectivity and development degree of the internal crack network in rocks are continuously enhanced, and the overall mechanical properties of rocks are eventually deteriorated.

Under the same number of freeze–thaw cycles, the AE *Ib*_1_ and *Ib*_2_ values of sandstone show an increasing trend when the freeze–thaw duration is extended by 1 h, indicating that the freeze–thaw duration exerts a regulatory effect on the damage evolution of rocks. On the one hand, a longer freezing and thawing time further reduces the unfrozen water content inside rocks and significantly increases the action time and intensity of frost-heaving force. On the other hand, the repeated water–ice phase transition promotes the more sufficient propagation and coalescence of micro-cracks and pores, leading to a higher development degree of the initial crack network inside rocks. The intensive development and extensive connectivity of micro-defects result in more active micro-crack activities during the failure process of rocks, thus showing a higher AE *Ib* value, which also confirms that the freeze–thaw duration is a key controlling factor for aggravating rock damage.

With the increase in the number and duration of freeze–thaw cycles, the internal microstructure of rocks undergoes irreversible deterioration, the development degree and connectivity of micro-pores and micro-cracks are continuously improved, and the freeze–thaw damage effect is continuously accumulated. These microstructural changes are ultimately reflected in the decrease in peak stress in macroscopic mechanical properties. The AE *Ib* value can be used as an important characterization parameter reflecting the degree of freeze–thaw damage of rocks. Its evolution law can effectively reveal the regulatory mechanism of freeze–thaw cycles on the internal micro-cracks and pore structure of rocks, providing an important microscopic basis for the stability evaluation and disaster early warning of freeze–thaw rock masses.

## 5. Freeze–Thaw Creep Damage Constitutive Model

### 5.1. Model Establishment

Under the action of freeze–thaw cycles, the internal structure of sandstone will be damaged to varying degrees due to the frost-heaving force caused by water–ice phase transition and the stress fluctuation caused by the temperature difference between the inside and outside of the rock. Studies have shown [24] that this damage will lead to the gradual degradation of the physical and mechanical properties of sandstone. Therefore, when constructing the creep constitutive model of sandstone, the freeze–thaw damage factor caused by freeze–thaw cycles must be taken into account.

Lemaitre [37] proposed the strain equivalence hypothesis: replacing the total stress with the effective stress, the strain obtained from the undamaged material is equivalent to the strain generated by the total stress acting on the damaged material. After a series of derivations, the damage variable expression based on elastic modulus is obtained.

Based on the creep characteristics of sandstone under different stress levels, a creep model of sandstone after freeze–thaw cycles was established on the basis of the nonlinear viscoplastic body. To realize the response of the model to the two key factors of “number of cycles” and “cycle duration” in freeze–thaw cycles and accurately reflect their influences on the creep characteristics of sandstone, the freeze–thaw damage factor D defined above was introduced.

(4) $$D_{E}=1-\frac{E^{\prime }}{E}$$

where E is the elastic modulus of the material in the undamaged state; $E^{\prime }$ is the damaged elastic modulus.

The freeze–thaw damage variables of sandstone after different freeze–thaw cycles were calculated by Equation (4), as shown in Table 3.

The characteristic parameters of some components of the model (including elastic modulus and viscosity coefficient) were modified by this variable. After modification, the model components can effectively reflect the damage and degradation effect of sandstone caused by freeze–thaw action, thus being more in line with the actual creep behavior of sandstone after freeze–thaw.

**Figure 15:**
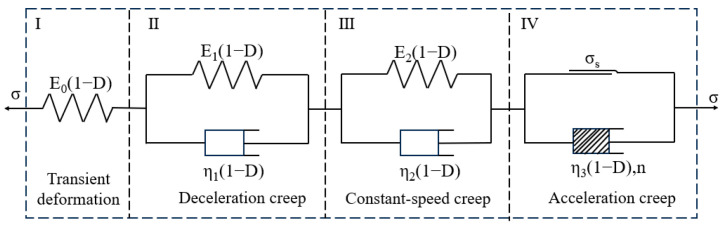
Nonlinear viscoelastic creep model of sandstone after F–T cycles [38].

As shown in Figure 15, when the creep stress level $\sigma <\sigma_{L}$ (long-term strength), only three parts (I, II, III) participate in the creep, which correspond to eristics of instantaneous elastic deformation, decelerating creep stage, and steady creep stage in the creep process. The creep model is a five-element linear viscoelastic model, and its corresponding state equation is the following:

(5) $$\left\{\begin{array}{l} \sigma_{0}=E_{0}(1-D)\varepsilon_{0}\\ \sigma_{1}=E_{1}(1-D)\varepsilon_{1}+\eta_{1}(1-D)\varepsilon_{1}^{\prime }\\ \sigma_{2}=E_{2}(1-D)\varepsilon_{2}+\eta_{2}(1-D)\varepsilon_{2}^{\prime }\\ \sigma =\sigma_{0}=\sigma_{1}=\sigma_{2}\\ \varepsilon =\varepsilon_{0}+\varepsilon_{1}+\varepsilon_{2} \end{array}\right.$$

where σ and ε are the total stress and total strain of the creep model, respectively; $\sigma_{0}$, $\sigma_{1}$, $\sigma_{2}$ are the stresses of parts I, II, III, respectively; $\varepsilon_{0}$, $\varepsilon_{1}$, $\varepsilon_{2}$ are the strains of parts I, II, III, respectively; $E_{0}$, $E_{1}$, $E_{2}$ and $\eta_{1}$, $\eta_{2}$ are the elastic and viscous parameters of the material, respectively.

According to Equation (5), the constitutive equation of the five-element viscoelastic creep model can be obtained as

(6) $$\begin{array}{l} \varepsilon^{\prime \prime }+\left(\frac{E_{1}}{\eta_{1}}+\frac{E_{2}}{\eta_{2}}\right)\varepsilon^{\prime }+\frac{E_{1}E_{2}}{\eta_{1}\eta_{2}}\varepsilon =\\ \frac{1}{E_{0}(1-D)}\sigma^{\prime \prime }+\left[\frac{E_{1}}{E_{0}\eta_{1}(1-D)}+\frac{\eta_{1}+\eta_{2}}{\eta_{1}\eta_{2}(1-D)}+\frac{E_{2}}{E_{0}\eta_{2}(1-D)}\right]\sigma^{\prime }+\left(\frac{E_{0}E_{1}+E_{1}E_{2}+E_{0}E_{2}}{E_{0}\eta_{1}\eta_{2}(1-D)}\right)\sigma \end{array}$$

When t = 0, substituting the following initial conditions

(7) $$\left\{\begin{array}{l} \varepsilon_{i}^{\prime }(0)=\varepsilon_{i}^{\prime \prime }(0)=0\\ \sigma_{0}^{\prime }(0)=\sigma_{i}^{\prime \prime }(0)=0\\ \left(i=1,2,3\right) \end{array}\right.$$

The creep equation can be obtained as

(8) $$\varepsilon =\frac{\sigma }{E_{0}(1-D)}+\frac{\sigma }{E_{1}(1-D)}\left(1-e^{-\frac{E_{1}}{\eta_{1}}t}\right)+\frac{\sigma }{E_{2}(1-D)}\left(1-e^{-\frac{E_{2}}{\eta_{2}}t}\right)$$

When the creep stress level $\sigma \geq \sigma_{s}$, it corresponds to the deformation characteristics of instantaneous elastic deformation, decelerating creep stage, steady creep stage, and accelerating creep stage in the creep process, that is, all basic components in the creep model produce deformation. At this time, the creep model is a seven-element linear viscoelastic model, and its corresponding state equation is

(9) $$\left\{\begin{array}{l} \sigma_{0}=E_{0}(1-D)\varepsilon_{0}\\ \sigma_{1}=E_{1}(1-D)\varepsilon_{1}+\eta_{1}(1-D)\varepsilon_{1}^{\prime }\\ \sigma_{2}=E_{2}(1-D)\varepsilon_{2}+\eta_{2}(1-D)\varepsilon_{2}^{\prime }\\ \sigma_{3}=\sigma_{s}+\eta_{3}(1-D)\varepsilon_{3}^{\prime }/(nt^{n-1})\\ \sigma =\sigma_{0}=\sigma_{1}=\sigma_{2}=\sigma_{3}\\ \varepsilon =\varepsilon_{0}+\varepsilon_{1}+\varepsilon_{2}+\varepsilon_{3} \end{array}\right.$$

where $\sigma_{3}$ is the stress of part IV; $\sigma_{s}$ is the long-term strength of the rock; $\varepsilon_{3}$ is the strain of part IV; $\eta_{3}$ is the viscous parameter of the material; n is the creep exponent, reflecting the speed of the accelerating creep rate of the rock.

Based on Equation (6), the constitutive equation for the seven-element viscoelastic creep model can be obtained as

(10) $$\begin{array}{l} \varepsilon^{\prime \prime }+\left(\frac{E_{1}}{\eta_{1}}+\frac{E_{2}}{\eta_{2}}\right)\varepsilon^{\prime }+\frac{E_{1}E_{2}}{\eta_{1}\eta_{2}}\varepsilon =\\ \frac{1}{E_{0}(1-D)}\sigma^{\prime \prime }+\left[\frac{E_{1}}{E_{0}\eta_{1}(1-D)}+\frac{\eta_{1}+\eta_{2}}{\eta_{1}\eta_{2}(1-D)}+\frac{E_{2}}{E_{0}\eta_{2}(1-D)}\right]\sigma^{\prime }\\ +\left(\frac{E_{0}E_{1}+E_{1}E_{2}+E_{0}E_{2}}{E_{0}\eta_{1}\eta_{2}(1-D)}\right)\sigma +\frac{nt^{n-1}}{\eta_{3}(1-D)}\sigma^{\prime }+\frac{n(n-1)t^{n-2}(\sigma -\sigma_{s})}{\eta_{3}(1-D)}\\ +\left(\frac{E_{1}}{\eta_{1}}+\frac{E_{2}}{\eta_{2}}\right)\frac{nt^{n-1}(\sigma -\sigma_{s})}{\eta_{3}(1-D)}+\frac{E_{1}E_{2}}{\eta_{1}\eta_{2}}\int \frac{nt^{n-1}(\sigma -\sigma_{t})}{\eta_{3}(1-D)}dt \end{array}$$

When t = 0 and applying the initial conditions from Equation (9) yields

(11) $$\varepsilon =\frac{\sigma }{E_{0}(1-D)}+\frac{\sigma }{E_{1}(1-D)}\left(1-e^{-\frac{E_{1}}{\eta_{1}}t}\right)+\frac{\sigma }{E_{2}(1-D)}\left(1-e^{-\frac{E_{2}}{\eta_{2}}t}\right)+\frac{\sigma -\sigma_{s}}{\eta_{3}(1-D)}t^{\prime \prime }$$

Therefore, the creep equation for the sandstone creep model after freeze–thaw cycles is

(12) $$\varepsilon =\left\{\begin{array}{ll} \frac{\sigma }{E_{0}(1-D)}+\frac{\sigma }{E_{1}(1-D)}\left(1-e^{-\frac{E_{1}}{\eta_{1}}t}\right)+\frac{\sigma }{E_{2}(1-D)}\left(1-e^{-\frac{E_{2}}{\eta_{2}}t}\right)\; & \left(\sigma <\sigma_{s}\right)\\ \frac{\sigma }{E_{0}(1-D)}+\frac{\sigma }{E_{1}(1-D)}\left(1-e^{-\frac{E_{1}}{\eta_{1}}t}\right)+\frac{\sigma }{E_{2}(1-D)}\left(1-e^{-\frac{E_{2}}{\eta_{2}}t}\right)+\frac{\sigma -\sigma_{s}}{\eta_{3}(1-D)}t^{n}\; & \left(\sigma \geq \sigma_{s}\right) \end{array}\right.$$

### 5.2. Model Validation

**Figure 16:**
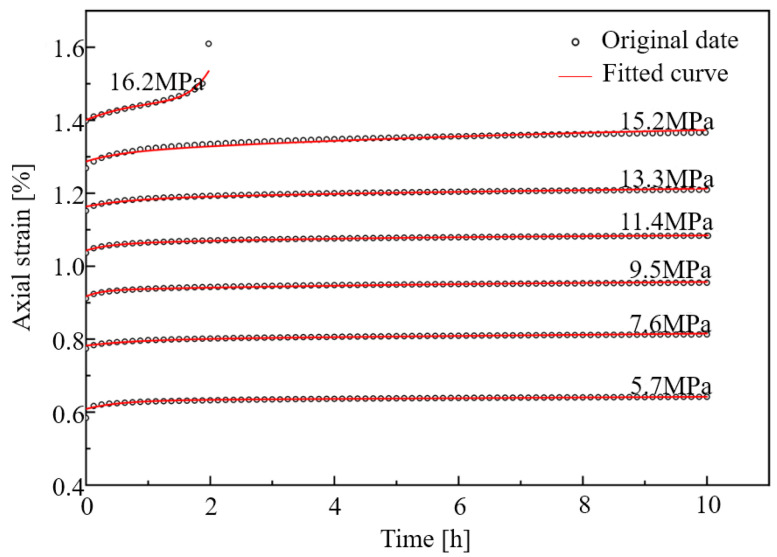
Creep axial strain–time data and fitted curve of sandstone after 50 cycles of F-1 h and T-1 h.

To verify the applicability of the freeze–thaw damage creep model, combined with the creep test results, model parameter calculation was carried out by selecting an appropriate mathematical model identification method. In this paper, the Levenberg–Marquardt method [39] was adopted for parameter fitting of the creep model. As a commonly used method for least squares estimation of regression parameters in nonlinear regression, this method has a core advantage in that it can integrate the characteristics of the steepest descent method and the linearization method, balancing fitting efficiency and accuracy. Based on the creep test data under different cyclic freeze–thaw conditions, the creep results of the specimens were fitted and analyzed. Only the model parameters and fitting results of the specimens subjected to 50 cycles of 1 h freezing/1 h thawing are presented herein, as detailed in Figure 16 and Table 4, Table 5 and Table 6.

The results indicate that this model exhibits high consistency with the test data, with the mean fitting degree reaching over 0.956, which demonstrates that the model can well characterize the creep deformation characteristics of the specimens after different cyclic freeze–thaw conditions [40]. The established creep model is expected to provide a reference for the long-term stability analysis of freeze–thaw geotechnical engineering.

## 6. Discussion

Compared with traditional freeze–thaw research, short-term freeze–thaw is characterized by short duration and high frequency of cycles. To deeply explore the internal damage mechanism of sandstone, scanning electron microscopy (SEM) was used to analyze the microscopic characteristics of sandstone, revealing the essential causes of rock damage under different conditions.

**Figure 17:**
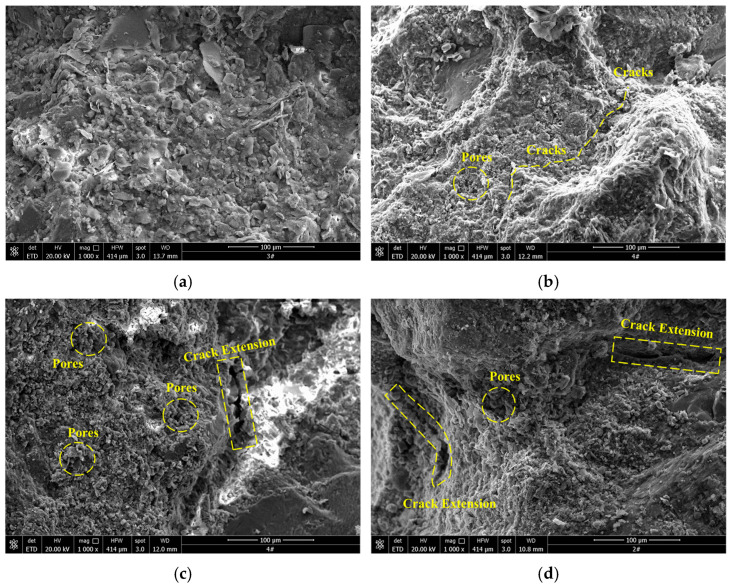
SEM images of sandstone after different freeze–thaw cycles for F-1 h and T-1 h. (**a**) 0 cycles; (**b**) 10 cycles; (**c**) 30 cycles; (**d**) 50 cycles.

**Figure 18:**
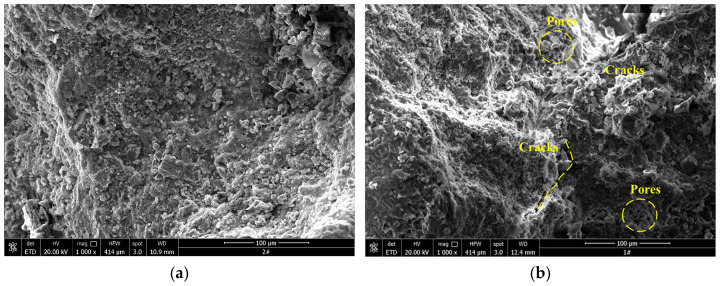

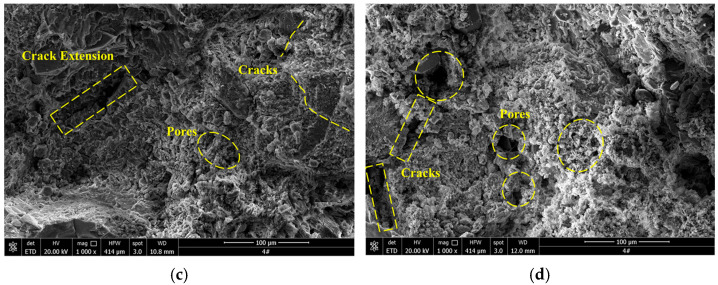
SEM images of sandstone after different freeze–thaw cycles for F-2 h and T-2 h. (**a**) 0 cycles; (**b**) 10 cycles; (**c**) 30 cycles; (**d**) 50 cycles.

Figure 17 and Figure 18 show the SEM images of sandstone after different freeze–thaw cycles. The results indicate that after 50 cycles of F-2 h, T-2 h, the fracture width of sandstone reaches 60–80 μm, which is 1.7 times that of F-1 h, T-1 h sandstone (30–40 μm). Moreover, the proportion of through fractures increases from 12% to 45%, forming a “main fracture–branch fracture” network. This structural difference directly leads to the differentiation of macro and micro perspectives [41].

With the extension of freeze–thaw duration, the meso-structure of white sandstone gradually transforms from dense and ordered to loose and fragmented. As shown in Figure 17b and Figure 18b, the specimens without cyclic freeze–thaw (CFT) treatment only contain a small number of micro-pores and micro-fissures induced by water saturation and hydrolysis. These initial defects reduce the cementation strength between mineral particles, which is consistent with the core viewpoint proposed by SUN et al. [42] that changes in pore characteristics affect rock mechanical properties, and meanwhile create conditions for subsequent frost heaving. When the number of CFT cycles reaches 10, the number of micro-fissures and micro-pores inside the specimens increases significantly, indicating that at the initial stage of CFT, the frost-heaving force induced by water–ice phase transition in pores has begun to drive the propagation of initial micro-defects, promoting the initiation of new micro-fissures and micro-pores [43].

When the number of cyclic freeze–thaw (CFT) cycles reaches 30, the size of pores expands significantly with the occurrence of cluster-like aggregation, and the fissures also show a continuous propagation trend. As shown in Figure 17c,d and Figure 18c,d, this indicates that with the accumulation of CFT cycles, the freeze–thaw damage effect is continuously intensified. The repeated frost heaving–thawing shrinkage caused by water–ice phase transition promotes the continuous connection and merging of existing micro-defects, bringing the development of pores and fissures into an accelerated stage [44]. After 50 CFT cycles, large pores are formed inside the specimens, the width and length of fissures increase substantially, and some mineral particles are fractured, resulting in a sharp decline in the structural stability and strength of the rock. This is consistent with the viewpoint proposed by SONG et al. [45,46] that the evolution of unfrozen water content during the freeze–thaw process dominates the changes in rock mechanical parameters. A comparison of the two groups of specimens, namely the 1 h freezing/1 h thawing and 2 h freezing/2 h thawing specimens, shows that the latter exhibits denser pore development and faster fissure propagation rate under the same number of CFT cycles.

Based on the comprehensive analysis of SEM images, when the internal temperature of saturated specimens drops to the freezing point, the water in the pores and fractures undergoes water–ice phase transition, and the frost-heaving force generated by freezing expansion acts continuously on the walls of pores and fractures. Due to the longer duration of a single freeze–thaw cycle of the 2 h freezing/2 h thawing specimens, the frost-heaving force exerts a more sustained effect, resulting in a significantly higher degree of internal damage compared with the 1 h freezing/1 h thawing group. When the temperature rises, ice melts into water and its volume contracts, but the already expanded pores and fractures cannot recover. The cementitious materials inside the rock are gradually lost during the seepage process, which further exacerbates pore development [47]. With the increase in the number of freeze–thaw cycles, discrete pores gradually aggregate into a clustered pore network. Pores between clusters interconnect to form larger-scale cavities, and fractures continue to extend and propagate, ultimately leading to severe deterioration of the internal rock structure and a substantial decline in macroscopic mechanical properties.

Short-term freeze–thaw cycles are characterized by a short freeze–thaw period and high cycle frequency. By altering the microstructural morphology of rocks and aggravating the accumulation of internal damage, they further affect the creep characteristics of white sandstone. This study deepens the damage response mechanism of rocks under short-term freeze–thaw action, providing a certain scientific reference for the safety design and disaster prevention of related geotechnical engineering projects. Future research will carry out in-depth exploration focusing on the prediction of long-term deformation laws of freeze–thawed rock masses, as well as the dynamic evaluation technology for rock mass stability based on acoustic emission (AE) characteristics.

## 7. Conclusions

This paper investigates the creep characteristics, acoustic emission (AE) characteristics, and damage evolution laws of white sandstone under short-term freeze–thaw action, and establishes a creep constitutive model considering the freeze–thaw damage effect. The main conclusions are drawn as follows:

(1) The duration and frequency of short-term freeze–thaw cycles exert a significant aggravating effect on the damage of specimens. The mass loss rate, longitudinal wave velocity reduction rate, and porosity growth rate of sandstone all exhibit an increasing trend with the rise in freeze–thaw cycles. Moreover, when the freeze–thaw duration is extended by 1 h, the growth rate of the above damage indicators becomes more pronounced. After 50 freeze–thaw cycles, the mass loss rate of the 2 h freezing/2 h thawing specimens reaches 1.48%, corresponding to 2.03 times that of the 1 h freezing/1 h thawing specimens (0.73%); the longitudinal wave velocity reduction rate hits 58.49%, which is 1.78 times that of the 1 h freezing/1 h thawing specimens (32.95%); the porosity growth rate amounts to 44.32%, equivalent to 1.64 times that of the 1 h freezing/1 h thawing specimens (27.09%). This indicates that extending the duration of a single freeze–thaw cycle can accelerate the development of internal pores and the coalescence of fractures in rocks, significantly impairing their structural integrity.
(2) The creep curves of all specimens exhibit the three-stage characteristic of deceleration–steady–acceleration. The non-failure stress stage (30–80% *R_c_*) only involves the deceleration and steady creep stages, while the failure stress stage (≥80% *R_c_*) undergoes the complete three-stage evolution. Under different freeze–thaw durations and cycle numbers, the instantaneous axial strain of the specimens increases with the elevation of stress levels. At the same cycle number, the instantaneous axial strain of the 2 h freezing/2 h thawing specimens is consistently larger than that of the 1 h freezing/1 h thawing specimens. For instance, after 50 cycles, the instantaneous axial strain of the 2 h freezing/2 h thawing specimens under the sixth stress level reaches 1.484%, which is 1.17 times that of the 1 h freezing/1 h thawing specimens (1.269%). The long-term strength of the specimens decreases with the increase in freeze–thaw cycle numbers and duration, and the freeze–thaw duration is the key controlling factor for strength attenuation. After 50 cycles, the long-term strength of the 1 h freezing/1 h thawing and 2 h freezing/2 h thawing specimens decreases by 40.15% and 55.60%, respectively, indicating that prolonged freeze–thaw promotes damage accumulation by extending the duration of crystalline expansion, resulting in a significant decline in the deformation resistance of rocks.
(3) The acoustic emission (AE) event rate is highly coupled with the creep stage. It exhibits a three-stage pattern of increase–decrease–stabilization in the non-failure stress stage and a typical U-shaped evolution characteristic in the failure stress stage. In the accelerating creep stage, due to the coalescence of macroscopic cracks, the AE event rate surges sharply to a peak value (up to more than 1000 events/s), providing a clear signal indicator for rock mass instability. The cumulative AE event rate decreases significantly with the increase in freeze–thaw cycle numbers and duration. After 50 cycles, the cumulative AE event rate of the 1 h freezing/1 h thawing specimens decreases by 35% compared with that of the control group of unfrozen–thawed specimens, while the rate of the 2 h freezing/2 h thawing specimens decreases by 70%. At the same cycle number, the AE activity intensity of the 2 h freezing/2 h thawing specimens is consistently lower than that of the 1 h freezing/1 h thawing specimens, and the moment of abrupt surge in the event rate in the failure stress stage occurs earlier. This reflects that prolonged freeze–thaw results in a more developed initial crack network in the rock, leading to fewer new crack initiations during subsequent loading, which indicates that substantial damage occurs at a lower stress level and the rate of damage evolution is faster.
(4) The AE *Ib* value evolves regularly with freeze–thaw duration and the number of freeze–thaw cycles. Under the same freeze–thaw duration, the AE *Ib*_1_ and *Ib*_2_ values exhibit a continuous decreasing trend with the increase in the number of freeze–thaw cycles. Under the same number of freeze–thaw cycles, the AE *Ib*_1_ and *Ib*_2_ values of sandstone show an increasing trend when the freeze–thaw duration is extended by 1 h. This indicates that extending the freeze–thaw duration and increasing the number of freeze–thaw cycles aggravate the development of internal cracks in rocks and accelerate the rate of damage evolution.
(5) Based on the seven-element viscoelastic model, a freeze–thaw damage factor D considering both freeze–thaw duration and number of cycles was introduced to construct a short-term freeze–thaw creep damage constitutive model. The average goodness-of-fit of the model reaches 0.964, and the strain prediction error is controlled within 3.19%. The maximum relative errors of sandstone after 50 cycles under 1 h freezing/1 h thawing and 2 h freezing/2 h thawing conditions are 2.55% and 3.19%, respectively. It can well characterize the full-stage creep mechanical behavior of sandstone after freeze–thaw degradation, providing a theoretical model reference for the long-term deformation prediction and stability assessment of geotechnical engineering in short-term freeze–thaw zones.

## Figures and Tables

**Table 1:** Graded loading creep test protocol.

Freeze–Draw Duration	Cycles	*R_c_* [MPa]	Loading Stress Level [MPa]						
			Level 1	Level 2	Level 3	Level 4	Level 5	Level 6	Level 7
Unfreeze–thaw	0	28.25	9	12	15	18	21	24	25.41
F-1 h, T-1 h	10	27.72	8.4	11.2	14.0	16.8	19.6	22.4	23.80
	20	27.13	8.1	10.8	13.5	16.2	18.9	21.6	23.00
	30	24.16	7.5	10.0	12.5	15.0	17.5	20.0	21.30
	40	22.72	6.9	9.2	11.5	13.8	16.1	18.4	19.54
	50	18.76	5.7	7.6	9.5	11.4	13.3	15.2	16.20
F-2 h, T-2 h	10	27.53	8.4	11.2	14.0	16.8	19.6	22.4	23.80
	20	22.32	6.6	8.8	11.0	13.2	15.4	17.6	18.72
	30	18.18	5.4	7.2	9.0	10.8	12.6	14.4	15.45
	40	17.45	5.1	6.8	8.5	10.2	11.9	13.6	14.83
	50	14.34	4.5	6.0	7.5	9.0	10.5	12.0	12.19

**Table 2:** Instantaneous axial strain of sandstone under different freeze–thaw conditions.

Loading Level	Instantaneous Axial Instantaneous Strain [%]											Instantaneous Axial Strain Rate [%]				
	Unfrozen–Thawed	F-1 h, T-1 h					F-2 h, T-2 h									
	0	10	20	30	40	50	10	20	30	40	50	10	20	30	40	50
1	0.411	0.450	0.478	0.507	0.543	0.584	0.475	0.492	0.510	0.558	0.598	9.44	0.61	0.61	2.75	2.35
2	0.554	0.597	0.618	0.667	0.704	0.774	0.638	0.643	0.693	0.748	0.815	7.70	3.20	3.83	6.23	5.25
3	0.679	0.712	0.739	0.793	0.838	0.912	0.740	0.772	0.813	0.892	0.993	3.96	4.41	2.62	6.41	8.90
4	0.785	0.815	0.838	0.900	0.971	1.036	0.837	0.886	0.938	1.023	1.166	2.64	5.74	4.21	5.28	12.50
5	0.870	0.912	0.935	0.994	1.104	1.152	0.929	0.997	1.057	1.144	1.324	1.82	6.70	6.29	3.58	14.94
6	0.970	1.009	1.037	1.097	1.235	1.269	1.014	1.114	1.191	1.271	1.484	0.56	7.41	8.58	2.92	16.95
7		1.071	1.107	1.202		1.399	1.089	1.071				1.66				

**Table 3:** Variables of F–T damage in sandstone under different cyclic conditions.

T/h	D				
	N = 10	N = 20	N = 30	N = 40	N = 50
1	0.091	0.016	0.036	0.042	0.054
2	0.083	0.024	0.051	0.057	0.068

**Table 4:** Comparative data of model validation for sandstone after 50 freeze–thaw cycles under two conditions.

Freeze–Thaw Time	Stress Level [MPa]	Final Test Strain [%]	Model Prediction of Strain [%]	Absolute Error [%]	Relative Error [%]
F-1 h, T-1 h	5.7	0.582	0.575	0.007	1.20
	7.6	0.763	0.758	0.005	0.65
	11.4	1.153	1.142	0.011	0.95
	15.2	1.735	1.722	0.013	0.75
	16.2	3.056	2.978	0.078	2.55
F-2 h, T-2 h	4.5	0.612	0.605	0.007	1.14
	6.0	0.815	0.808	0.007	0.86
	9.0	1.326	1.314	0.012	0.90
	10.5	1.688	1.670	0.018	1.07
	12.0	2.885	2.793	0.092	3.19

**Table 5:** Creep model parameters of specimens subjected to 50 cycles of F-1 h and T-1 h.

N	σ	*E*_0_	*E*_1_	*E*_2_	*η*_1_	*η*_2_	*η*_3_	n	*R* ^2^
50	5.7	14.103	361.259	302.867	199.146	8820.083			0.866
	7.6	14.632	117.105	679.859	7007.389	582.794			0.972
	9.5	15.594	823.803	429.034	207.460	3873.028			0.949
	11.4	16.455	615.166	968.365	3358.333	363.897			0.964
	13.3	18.121	404.798	989.181	5284.667	705.779			0.992
	15.2	17.785	232.664	948.275	2316.930	523.941			0.998
	16.2	17.202	19.899	379.873	4734.643	338.549	4098.723	6.811	0.957

**Table 6:** Creep model parameters of specimens subjected to 50 cycles of F-2 h and T-2 h.

N	σ	*E*_0_	*E*_1_	*E*_2_	*η*_1_	*η*_2_	*η*_3_	n	*R* ^2^
50	4.5	15.100	270.479	245.412	37.392	1373.086			0.988
	6.0	15.165	540.351	293.591	37.708	937.426			0.938
	7.5	15.521	659.823	353.575	61.530	1312.753			0.994
	9.0	15.708	570.754	379.907	415.903	6404.167			0.979
	10.5	16.229	592.010	375.307	304.497	3562.389			0.984
	12.0	17.048	154.656	999.999	280.624	957.324	3956.835	2.239	0.931

## Data Availability

The original contributions presented in this study are included in the article. Further inquiries can be directed to the corresponding authors.
